# Short Forms of the German Revised Children's Anxiety and Depression Scale (RCADS)–Validation and Normative Data of the 11‐ and 25‐Item Versions

**DOI:** 10.1002/mpr.70022

**Published:** 2025-04-26

**Authors:** Susanne Grothus, Ariane Sommer, Benedikt B. Claus, Lorin Stahlschmidt, Lea Höfel, Bruce F. Chorpita, Julia Wager

**Affiliations:** ^1^ German Paediatric Pain Centre Children's and Adolescents' Hospital Datteln Germany; ^2^ Department of Children's Pain Therapy and Paediatric Palliative Care Faculty of Health School of Medicine Witten/Herdecke University Witten Germany; ^3^ PedScience Research Institute Datteln Germany; ^4^ Center for Pain Therapy for Young People Garmisch‐Partenkirchen Germany; ^5^ Department of Psychology University of California Los Angeles California USA

**Keywords:** anxiety, depression, psychometric properties, RCADS short version, youth

## Abstract

**Objectives:**

This study assesses the reliability and validity of two short forms of the German Revised Children’s Anxiety and Depression Scale (RCADS 11‐ and 25‐item versions) and provides normative data.

**Methods:**

Data were collected from a representative sample of *N* = 1562 German schoolchildren and *N* = 757 pediatric chronic pain patients (ages 8–17).

**Results:**

Cronbach's α demonstrated acceptable to good internal consistency for the total score as well as the depression and anxiety scales. Confirmatory factor analysis (CFA) demonstrated acceptable to good model fit for both a 2‐factor structure (RCADS‐11) and a higher‐order structure (RCADS‐25). Multi‐group CFAs demonstrated similar model structures across the school and pediatric chronic pain samples. Convergent validity was supported by moderate to high negative correlations with health‐related quality of life and a high positive correlation with functional impairment. Girls—and to some extent, adolescents – scored significantly higher on anxiety and depression scales. The short versions demonstrated excellent agreement with the original 47‐item RCADS (0.78 ≤ Cohen’s *κ* ≤ 1.0). German normative data are provided.

**Conclusion:**

The short versions of the German RCADS are reliable and valid instruments for assessing anxiety and depressive symptoms in children and adolescents.

## 1 Introduction

Anxiety and depression pose serious mental health risks in childhood and adolescence (Beesdo et al. 2009; Kessler and Bromet 2013). In the US, prevalence rates prior to the Covid‐19 pandemic reached up to 20% for anxiety (Ghandour et al. 2019; Kessler et al. 2012) and up to 11% for depression (Avenevoli et al. 2015; Ghandour et al. 2019; Mojtabai et al. 2016), with rates increasing significantly during the pandemic (Wang et al. 2022). A recent systematic review estimated a pooled global prevalence of 31% for emotional problems post‐pandemic (Deng et al. 2023). In Germany, prevalence rates for depression among children and adolescents returned to pre‐pandemic levels of around 15% (Klasen et al. 2016; Ravens‐Sieberer et al. 2023), whereas anxiety symptoms rose from 15% (Klasen et al. 2016) to 25%, even after the pandemic had subsided (Ravens‐Sieberer et al. 2023).

If not identified and treated at an early stage, anxiety and depression can persist into adulthood (Fichter et al. 2009; Johnson et al. 2018; Pine et al. 1998). Early detection and intervention using a standardized assessment tool for anxiety and depression symptoms is crucial to prevent the manifestation of these symptoms and related impairments. The Revised Children's Anxiety and Depression Scale (RCADS; Chorpita et al. 2000) is a widely used, no‐cost screening instrument that measures anxiety and depression symptoms in children and adolescents aged eight to 18 years through self‐report. Its psychometric properties have been well established across diverse populations and languages. The original 47‐item RCADS, including the German version (Grothus et al. 2023; Stahlschmidt et al. 2019), demonstrates good reliability and validity in both community samples (Chorpita et al. 2000; Donnelly et al. 2019; Esbjørn et al. 2012; Fard et al. 2021; Fontana et al. 2019; Giannopoulou et al. 2022; Kösters et al. 2015; Lu et al. 2021; Muris et al. 2002; Petrič et al. 2024; Ross et al. 2002) and clinical samples (Chorpita et al. 2005; Gormez et al. 2017; Stahlschmidt et al. 2019). The questionnaire has also indicated high cross‐cultural validity (Stevanovic et al. 2017), which ensures its comparability across countries and ethnicities. As of 2025, the RCADS 47‐item version is freely available in 30 different languages, including German (see https://rcads.ucla.edu/versions). Despite the lower reliability of shorter versions (Piqueras et al. 2017), they are of interest as they reduce the burden on respondents, improving acceptance and participation rates, especially in epidemiological studies and primary care settings. In clinical settings, shorter instruments can be used for initial assessments and simplify the long‐term monitoring of emotional impairment. Ebesutani et al. (2012) developed a reliable and valid 25‐item short version of the RCADS which has been translated into 16 languages (see also https://rcads.ucla.edu/versions) and has been part of the National Institutes of Health Common Data Elements Initiative since July, 2020 (see https://grants.nih.gov/grants/guide/notice‐files/NOT‐MH‐20‐067.html). However, not all short versions have been formally validated (Carlander et al. 2024; Ebesutani et al. 2012; Klaufus et al. 2020; Lisøy et al. 2022; Young et al. 2021). Radez et al. (2021) developed an even shorter, 11‐item version for detecting anxiety and depressive symptoms in British adolescents aged 11–17 years, demonstrating promising psychometric properties in both community and clinical samples (Radez et al. 2021).

In Germany, neither the 25‐item nor the 11‐item RCADS versions have been examined psychometrically. Measurement invariance has not been tested, and no German normative data have been generated for these short versions. These steps are urgently needed to ensure the German short versions are comparable with data from other countries and languages.

This study therefore aims to: (a) examine the psychometric properties of the German RCADS‐25 and RCADS‐11, focusing on item properties, internal consistency, construct validity (including factorial validity including measurement invariance), convergent validity, and known‐groups validity, (b) provide sex‐ and age‐specific as well as non‐specific gender normative data, and (c) compare the RCADS full version (RCADS‐47), RCADS‐25, and RCADS‐11 to assess whether the same constructs are captured and whether the three versions consistently identify the same individuals with normal or clinically relevant T‐scores.

## 2 Method

### 2.1 Study Design and Participants

The present study used data collected as part of the MeMaps project (“Chronische Schmerzen bei Kindern und Jugendlichen–multidimensionales Ergebnisqualitätsmaß und praxistaugliche Stratifizierungsstrategie”), which was approved by the Ethics Committee of the University Witten/Herdecke, Germany (Approval code 75/2019). Baseline data from a large, representative school sample (*N* = 1562) and a pediatric chronic pain sample from tertiary care (*N* = 757) were analyzed.

### 2.2 School Sample

Data collection took place in six different public schools: three secondary schools in October/November 2019, and three elementary schools in September 2021 (delayed due to the Covid‐19 pandemic). The secondary schools were representative of all possible school‐leaving qualifications in Germany. The study was presented to the headmasters, teachers, parents and students, and the project team distributed study information and consent forms to both parents and children. All children between the ages of eight and 17 who spoke sufficient German were eligible to participate, provided that parents and children gave their written consent/assent. In total, *N* = 2655 children and adolescents were eligible for participation, of which *N* = 1586 consented to participate. Of these, *N* = 1562 attended school on the days of data collection, resulting in a participation rate of 58.8% of the eligible population.

### 2.3 Pediatric Chronic Pain Sample

Additionally, MeMaps Project data for *N* = 757 pediatric chronic pain patients were used. Data were collected systematically from September 2019 to August 2021 in two tertiary pediatric chronic pain centers in Germany (German Pediatric Pain Center, Datteln, and the Center for Pain Therapy for Young People, Garmisch‐Partenkirchen). As in the schools, parents and children were informed orally and in writing about the study, and the conditions of participation were identical.

Demographic characteristics (e.g., age, sex ask with two response options: girl or boy) as well as psychological well‐being for both samples are displayed in Table 1.

**TABLE 1:** Demographics and psychological well‐being of school sample and pediatric chronic pain sample.

		School sample				Chronic pain sample			
		*n*	**%**	*M*	*SD*	*n*	**%**	*M*	*SD*
Sex	Female	818	52.4			552	72.9		
	Male	744	47.6			205	27.1		
Age				12.23	2.33			13.85	2.34
Functional impairment^†^				7.98	8.59			18.83	11.53
HRQoL				39.55	7.47			35.08	7.49

*Note:* HRQoL = Health‐Related Quality of Life, measured with KIDSCREEN‐10; *M* = mean; *SD* = standard deviation; *n* = sample size; ^†^measured with the Functional Disability Inventory (FDI).

For more details about these samples, see Grothus et al. (2024).

### 2.4 Measures

All data were collected electronically via self‐report using tablet PCs. Missing values were avoided by programming all responses as mandatory. Additional variables were collected within the MeMaps project but were not used in the present study.

#### 2.4.1 Revised Children's Anxiety and Depression Scale (RCADS‐25, RCADS‐11)

Anxiety and depressive symptoms were originally obtained using the validated German 47‐item version of the RCADS (Chorpita et al. 2000; Grothus et al. 2023; Stahlschmidt et al. 2019) and were subsequently shortened to the existing 25‐item (Ebesutani et al. 2012) and 11‐item versions (Radez et al. 2021). Table S1 and S2 in the Supporting Information S1 on which RCADS‐47 items are included in the 25‐ and 11‐item versions. The RCADS‐25 includes the same 10 items (e.g. “feels sad or empty”) that measure depressive symptoms (MDD) in the RCADS‐47, as well as 15 items (e.g. “worries something bad will happen”) that comprise a broad anxiety scale. Although three items were selected from each of the five anxiety subscales (general anxiety disorder (GAD), obsessive‐compulsive disorder (OCD), panic disorder (PD), separation anxiety disorder (SAD) and social phobia (SP)) in the RCADS‐47, the RCADS‐25 is not intended to assess specific anxiety subscales (Ebesutani et al. 2012). The RCADS‐11 comprises six items measuring anxiety symptoms (e.g. “suddenly feels scared for no reason”) and five items for depressive symptoms (e.g. “feels tired a lot”) (Radez et al. 2021). For both forms, all items were self‐reported on a 4‐point Likert scale from 0 (*never*) to 3 (*always*), with higher scores indicating more severe anxiety and/or depressive symptoms in children and adolescents eight to 17 years old (range_RCADS‐25_ = 0–75, range_RCADS‐11_ = 0–33) (Ebesutani et al. 2012; Radez et al. 2021). Reliability and factorial validity of these short forms have been demonstrated in school samples (Carlander et al. 2024; Ebesutani et al. 2012; Klaufus et al. 2020; Radez et al. 2021; Young et al. 2021) and clinical samples (Ebesutani et al. 2012; Radez et al. 2021).

#### 2.4.2 Functional Impairment

Functional impairment was assessed using the Functional Disability Inventory (FDI; Walker and Greene 1991). This tool evaluates impairment in 15 daily activities, asking children and adolescents aged eight to 18 years about their difficulties over the past four weeks (e.g. “being at school all day”). Responses are given on a five‐point Likert scale ranging from 0 (*no problems*) to four (*impossible*). A total score (range = 0–60) can be calculated, where higher scores indicate greater functional impairment. The German version of the FDI has demonstrated good reliability and validity (Offenbächer et al. 2016; Stahlschmidt et al. 2018). In the present sample, internal consistency of the FDI is high, with Cronbach's *α* = 0.90.

#### 2.4.3 Health‐Related Quality of Life

Health‐related quality of life (HRQoL) was assessed using the KIDSCREEN‐10 Index (Ravens‐Sieberer et al. 2010), a questionnaire for eight to 18 year‐old children and adolescents. It comprises 10 questions (e.g. “Have you had fun with your friends?”) self‐reported on a five‐point Likert scale from 1 (*never/not at all*) to 5 (*always/extremely*). A total score is calculated from the sum of all items (range = 10–50), where higher sum scores indicate better HRQoL. The KIDSCREEN‐10 has demonstrated good validity and reliability (Ravens‐Sieberer et al. 2010). In the present sample, internal consistency of the KIDSCREEN‐10 Index is satisfactory, with Cronbach's *α* = 0.85.

### 2.5 Data Analysis

Statistical analyses were conducted using IBM Statistical Package for Social Sciences (SPSS) version 28 for Windows. Confirmatory factor analyses (CFA) and Multigroup‐CFA (MG‐CFA) were performed using R version 4.1.2 (R Core Team 2022) and RStudio (RStudio Team 2020), with the *lavaan* package (Rosseel 2012). The significance level was set at *α* < 0.05. All analyses were based on data from the school sample; for the MG‐CFA, data from the chronic pain sample were also included.

To avoid α‐error accumulation due to multiple testing, a Bonferroni correction was applied according to the number of tests performed and the local significance level was adjusted to tests of convergent validity and known‐groups validity (Curtin and Schulz 1998). Cohen's *d* and *κ* effect sizes are reported (Cohen 1992, 2013).

### 2.6 Item Properties and Internal Consistency

Item and scale statistics include item means, standard deviations, ranges, and corrected item‐total correlations. Internal consistency was assessed using Cronbach's *α*.

### 2.7 Construct Validity

Construct validity was tested in terms of factorial validity, convergent validity, and known‐groups validity.

**Factorial validity.** Confirmatory Factor Analysis (CFA) was conducted to examine the factor structure, model fit, and factor loadings. Based on previous research, two different models were tested against each other for all versions: a 1‐factor model (one overall internalizing factor with all items) and a 2‐factor model (separate anxiety and depression scales). For the RCADS‐25, a higher‐order model was also tested, in which three anxiety items were assigned to each of five first‐order anxiety factors (GAD, OCD, PD, SAD, and SP), which in turn loaded onto a second‐order anxiety factor. The 10 items measuring depressive symptoms loaded directly onto an MDD factor. For the CFA, the weighted least square mean and variance adjusted (WLSMV) estimation method was chosen for categorical data, which is a diagonally weighted least squares (DWLS) model estimation incorporating mean and variance‐adjusted test statistics. Robust fit indices and standardized factor loadings for each measurement model are reported. Model fit comparisons were based on two recommendations: (1) Beaujean's (2014) criteria, where better model fit is indicated by a Comparative Fit Index (CFI) and Tucker–Lewis Index (TLI) closer to 1.0, and a Root Mean Square Error of Approximation (RMSEA) and Standardized Root Mean Square Residual (SRMR) closer to 0.0, and (2) the more conservative criteria by Schermelleh‐Engel et al. (2003), where CFI and TLI values ≥ 0.95 indicate an acceptable fit, and ≥ 0.97 indicate a good fit; RMSEA values ≤ 0.08 are acceptable, and ≤ 0.05 are good; SRMR values ≤ 0.10 are acceptable, and ≤ 0.05 are good. Δχ^2^‐tests were used to test the comparative fit of different models.

Measurement invariance was tested for RCADS‐25 and for RCADS‐11 using the CFA model with the best fit. In a stepwise procedure with more restricted models, the original, single group solution (configural) model was compared with a model with fixed factor loadings across groups (metric model), and then to a model with fixed intercepts across groups (scalar model). Each model was compared with the previous, less restrictive model, following the criterion that a Δ CFI of 0.01 or less indicates an acceptable level of restriction (Brown et al. 2013; Hirschfeld and Brachel 2014; Vandenberg and Lance 2000).

**Convergent Validity.** To assess the convergent validity of the RCADS‐25 and RCADS‐11, since the data did not follow a normal distribution, τ‐correlations between the anxiety and depression scales and measures of HRQoL or functional impairment were calculated and classified as small (|τ| = 0.1), moderate (|τ| = 0.3), or large (|τ| = 0.5) (Cohen 2013).

**Known‐Groups Validity.** Independent sample *t*‐tests were calculated to test whether there were differences in anxiety and depression scores on the RCADS‐25 and RCADS‐11 based on age. The degrees of freedom were adjusted due to unequal variances indicated by the Levene test. Age‐related differences in the scales were analyzed separately for boys and girls using τ‐correlations. Cohen's *d* effect sizes are reported and interpreted as small (|*d*| = 0.2), moderate (|*d*| = 0.5), or large (|*d*| = 0.8) (Cohen 1992, 2013).

### 2.8 Generation of Normative Data

Age and sex differences in anxiety and depression were confirmed for both the RCADS‐25 and RCADS‐11, prompting the differentiation of normative data by five age groups (8–9, 10–11, 12–13, 14–15, and 16–17 years) and by sex. For non‐specific gender, normative data were stratified by age only. A scoring program for the calculation of RCADS‐25 T‐scores will be available under https://rcads.ucla.edu/scoringoverview. Moreover, percentiles were calculated for anxiety and depressions scales of the RCADS‐25 and RCADS‐11.

### 2.9 Comparison of RCADS Short Forms

To assess whether the different forms of the RCADS capture the same constructs, Kendall's τ‐b correlations were conducted between the MDD, Anxiety, and Total Scores of the RCADS full version (RCADS‐47), RCADS‐25, and RCADS‐11. χ^2^‐tests for associations between T‐scores of RCADS‐47, RCADS‐25, and RCADS‐11 were performed to determine whether the three versions are consistent in identifying the same individuals with normal versus clinically relevant values. T‐scores were categorized as normal (≤ 59) or clinically relevant (≥ 60). Cohens *κ* measured the level of observed agreement, classified as slight (*κ* = 0–0.20), fair (*κ* = 21–0.40), moderate (*κ* = 0.41–0.60), substantial (*κ* = 0.61–0.80), and perfect (*κ* = 0.81–1.0) (Landis and Koch 1977).

## 3 Results

### 3.1 Item and Scale Properties

RCADS‐25 and RCADS‐11 showed similar tendencies in terms of item and scale properties. Despite the item means in both versions having a right‐skewed distribution, with ranges from 0.25 (“has to think special thoughts to stop bad events”) to 1.37 (“worries something awful will happen to family”; RCADS‐25) and 0.29 (“trouble going to school”) to 0.98 (“feels tired a lot”; RCADS‐11), all items received responses across the full scale from 0 to 3. The Anxiety Scale had average item means of *M*_anxiety‐25_ = 0.65 and *M*_anxiety‐11_ = 0.56, whereas the Depression Scale item means were *M*_*MDD‐25*_ = 0.67 and *M*_*MDD‐11*_ = 0.66. Detailed item properties of the RCADS‐25 and RCADS‐11 are displayed in Table S1 and Table S2 in the Supporting Information S1. High reliability was observed for both the RCADS‐25‐Anxiety Score and the RCADS‐25‐MDD (Cronbach's *α* = 0.87) and the Total Score (Cronbach's *α* = 0.92). For the RCADS‐11, internal consistency was slightly lower, with *α*_MDD_ = 0.81, *α*_Anxiety_ = 0.79, and *α*_Total Score_ = 0.87 (acceptable to good). In general, reliability was higher in girls and older children (see Table 2).

**TABLE 2:** Cronbach's *α* of RCADS‐25 and RCADS‐11 for the school sample.

		RCADS‐25			RCADS‐11		
Group	*N*	Total Score	Anxiety	MDD	Total Score	Anxiety	MDD
Total sample	1562	0.92	0.87	0.87	0.87	0.79	0.81
Girls	818	0.93	0.88	0.89	0.89	0.81	0.84
Boys	744	0.89	0.83	0.81	0.81	0.72	0.69
8–9 years	214	0.89	0.84	0.76	0.78	0.73	0.55
10–11 years	420	0.92	0.88	0.87	0.87	0.79	0.79
12–13 years	404	0.93	0.88	0.88	0.89	0.82	0.83
14–15 years	392	0.92	0.87	0.88	0.88	0.79	0.84
16–17 years	132	0.92	0.86	0.89	0.89	0.80	0.83

*Note:* MDD = Major depressive disorder; *N* = sample size, RCADS = Revised Children's Anxiety and Depression Scale.

### 3.2 Construct Validity

Table 3 displays fit indices for all models of RCADS‐25 and RCADS‐11 tested in CFA. For both versions, the 2‐Factor model yielded a significantly better fit to the data than the 1‐Factor model. Additionally, for the RCADS‐25, the fit of the 2‐Factor model was slightly worse than the higher‐order model. In the best‐fitting models, all items loaded acceptably to highly on their respective factors, indicating a strong relationship between each item and its corresponding factor (ʎ_RCADS‐25_ = 0.59–0.89; ʎ_RCADS‐11_ = 0.65–0.86; see Table S1 and Table S2 Supporting Information S1).

**TABLE 3:** Model fit of all models tested in the CFA of RCADS‐25 and RCADS‐11.

		χ^2^	*df*	*p*	CFI	TLI	RMSEA	SRMR
RCADS‐25	1‐Factor model	2919.02	275	< 0.001	0.90	0.90	0.08	0.06
	2‐Factor model	2272.13	274	< 0.001	0.93	0.92	0.07	0.06
	Higher‐order model	1690.59	269	< 0.001	0.95	0.94	0.06	0.05
RCADS‐11	1‐Factor model	615.59	44	< 0.001	0.95	0.94	0.09	0.06
	2‐Factor model	380.23	43	< 0.001	0.97	0.96	0.07	0.04

*Note:* CFA = Confirmatory Factor Analysis; CFI = Comparative Fit Index; RMSEA = Root Mean Square Error of Approximation; SRMR = Standardized Root Mean Square Residual; TLI = Tucker‐Lewis Index.

### 3.3 Measurement Invariance

Given the optimal model fit in the CFA, the 2‐Factor model of RCADS‐11 and the higher‐order model of RCADS‐25 were further tested for measurement invariance using MG‐CFA between the healthy school sample and the pediatric chronic pain sample. Both models demonstrated strong fits for configural, metric, and scalar invariance, with Δ CFI ranging between −0.006 and ± 0.001. Thus, the factor structure, loadings, and intercepts can be considered equivalent across groups, allowing for the proposal of measurement invariance between a German community sample and a sample of pediatric chronic pain patients in tertiary care. Results for the MG‐CFA of RCADS‐25 and RCADS‐11 are summarized in Tables 4 and Table 5.

**TABLE 4:** Multi‐group CFA for the higher‐order model (RCADS‐25): school sample versus pediatric chronic pain patients.

Model	χ^2^	*df*	CFI	RMSEA	SRMR	Δ CFI
Single group solutions						
School sample	1096.65	269	0.95	0.06	0.05	
Chronic pain sample	1466.69	269	0.92	0.08	0.07	
Tests of invariance						
Configural	3125.77	538	0.94	0.06	0.06	—
Metric	2324.66	561	0.96	0.05	0.06	0.001
Scalar	3196.34	604	0.94	0.06	0.06	−0.001

*Note:* Δ CFI = difference between CFI of each model and the model with less restrictions (i.e., metric vs. configural and scalar vs. metric); CFA = Confirmatory Factor Analysis; CFI = Comparative Fit Index; RMSEA = Root Mean Square Error of Approximation; SRMR = Standardized Root Mean Square Residual.

**TABLE 5:** Multi‐group CFA for the two‐factor model (RCADS‐11): school sample versus pediatric chronic pain patients.

Model	χ^2^	*df*	CFI	RMSEA	SRMR	Δ CFI
Single group solution						
School sample	380.23	43	0.97	0.07	0.04	—
Chronic pain sample	309.80	43	0.97	0.09	0.05	—
Tests of invariance						
Configural	689.25	86	0.97	0.08	0.05	—
Metric	525.06	95	0.98	0.06	0.05	0.000
Scalar	970.79	115	0.96	0.08	0.05	−0.006

*Note:* Δ CFI = difference between CFI of each model and the model with less restrictions (i.e., metric vs. configural and scalar vs. metric); CFA = Confirmatory Factor Analysis; CFI = Comparative Fit Index; RMSEA = Root Mean Square Error of Approximation; SRMR = Standardized Root Mean Square Residual.

### 3.4 Convergent Validity

MDD, Anxiety, and the Total Score demonstrated significant moderate to strong negative correlations with the KIDSCREEN‐10 score for both the RCADS‐25 (−0.51 ≤ *τ* ≤ −0.39, *N* = 1562, *p* ≤ 0.001) and the RCADS‐11 (−0.52 ≤ *τ* ≤ −0.38, *N* = 1562, *p* ≤ 0.001), indicating that higher emotional distress was associated with lower HRQoL. Significant positive τ‐correlations between the FDI and both the RCADS‐25 (0.39 ≤ *τ* ≤ 0.48, *N* = 1562, *p* ≤ 0.001) and RCADS‐11 scales (0.37 ≤ *τ* ≤ 0.46, *N* = 1562, *p* ≤ 0.001) revealed a moderate association between anxiety and depressive symptoms and functional impairment (see Table 6).

**TABLE 6:** τ‐Correlations of the RCADS‐25 and RCADS‐11 with functional impairment and HRQoL.

	RCADS‐25			RCADS‐11		
	MDD	Anxiety	Total Score	MDD	Anxiety	Total Score
HRQoL	−0.51*	−0.39*	−0.48*	−0.52*	−0.38*	−0.50*
Functional impairment^†^	0.48*	0.39*	0.47*	0.46*	0.37*	0.46*

*Note:* *Significant at *p* ≤ 0.003 after Bonferroni correction. HRQoL = Health‐Related Quality of Life, measured with KIDSCREEN‐10; RCADS = Revised Children's Anxiety and Depression Scale; MDD Major depressive disorder; ^†^measured with Functional Disability Inventory.

### 3.5 Known‐Groups Validity

After Bonferroni correction, significant differences in the means of the MDD, Anxiety, and Total Scores were observed between girls and boys in both RCADS‐25 and RCADS‐11, with larger means in girls (see Table 7). With Cohen's d between −0.52 and −0.61, effect sizes are considered large for both instruments. Significant but weak τ‐correlations stratified by sex demonstrated a weak positive association between RCADS‐25 scales and age in girls (*τ*_MDD_ = 0.14, *τ*_Anxiety_ = 0.05, *τ*_Total Score_ = 0.10, *N* = 1562, *p* ≤ 0.001), and a weak negative association in boys (*τ*_MDD_ = −0.09, *τ*_Anxiety_ = −0.11, *τ*_Total Score_ = −0.11, *N* = 1562, *p* ≤ 0.001). For RCADS‐11, a significant weak positive association between the scales and age was found only in girls (*τ*_MDD_ = 0.19, *τ*_Anxiety_ = 0.08, *τ*_Total Score_ = 0.15, *N* = 1562, *p* ≤ 0.001) (see Table 8).

**TABLE 7:** Means and standard deviations for RCADS‐25 and RCADS‐11 scales.

		Total Sample		Girls *n* = 818		Boys *n* = 744		*t* [95% CI]	p*	Cohen's *d*
		*N* = 1562								
		*M*	*SD*	*M*	*SD*	*M*	*SD*			
RCADS‐25	MDD	6.73	5.42	8.05	5.98	5.27	4.28	−2.78 [−3.30; −2.26]	< 0.001	−0.53
	Anxiety	9.03	6.57	10.80	7.10	7.07	5.28	−3.73 [−4.35; −3.11]	< 0.001	−0.59
	Total Score	15.75	11.21	18.85	12.25	12.34	8.75	−6.51 [−7.56; −5.46]	< 0.001	−0.61
RCADS‐11	MDD	3.27	2.97	4.02	3.30	2.46	2.29	−1.56 [−1.84; −1,28]	< 0.001	−0.55
	Anxiety	3.37	3.16	4.12	3.49	2.54	2.49	−1.59 [−1.89; −1.29]	< 0.001	−0.52
	Total Score	6.64	5.62	8.14	6.25	4.99	4.25	−3.15 [−3.68; −2.63]	< 0.001	−0.59

*Note:* * Significant at *p* ≤ 0.003 after Bonferroni correction. *N* = sample size; *M* = mean; *SD* = standard deviation; RCADS = Revised Children's Anxiety and Depression Scale; MDD = Major depressive disorder; CI = Confidence Interval.

**TABLE 8:** τ‐Correlations of the RCADS‐25 and RCADS‐11 with age.

	RCADS‐25			RCADS‐11		
	MDD	Anxiety	Total Score	MDD	Anxiety	Total Score
Age girls (*n* = 818)	0.14*	0.05*	0.10*	0.19*	0.08*	0.15*
Age boys (*n* = 744)	−0.09*	−0.11*	−0.11*	−0.05	−0.05	−0.05

*Note:* *Significant at *p* ≤ 0.003 after Bonferroni correction. *n* = sample size; RCADS = Revised Children's Anxiety and Depression Scale; MDD = Major depressive disorder.

### 3.6 Generation of Normative Data

Means and standard deviations for the calculation of T‐Scores are provided for RCADS‐25 (see Supporting Information S1 Table S3) and RCADS‐11 (see Supporting Information S1 Table S4), stratified by age and sex, according to data from *N* = 1562 schoolchildren. For non‐specific gender T‐scores, means and standard deviations are calculated based solely on age (Supporting Information S1 Table S5 and S6). In addition, percentile scores are available in the Appendix (see Supporting Information S1 Tables S7 to S10).

### 3.7 Comparison of RCADS‐47, RCADS‐25, and RCADS‐11

There were significant high positive τ‐correlations between corresponding scales of RCADS‐47, RCADS‐25, and RCADS‐11, ranging from 0.66 to 0.87 (*N* = 1562, *p* ≤ 0.001). Significant positive τ‐correlations between non‐corresponding scales ranged from 0.51 to 0.81. In general, correlations between corresponding scales were higher than those between non‐corresponding scales in each version comparison. τ‐correlations are displayed in Table 9.

**TABLE 9:** τ‐Correlations between scales of RCADS‐47, RCADS‐25, and RCADS‐11.

		RCADS‐47			RCADS‐25		
		MDD	Anxiety	Total score	MDD	Anxiety	Total Score
RCADS‐25	MDD	1.00*	0.58	0.69	—	—	—
	Anxiety	0.55	**0.84**	0.81	—	—	—
	Total Score	0.77	0.79	**0.87**	—	—	—
RCADS‐11	MDD	**0.82**	0.54	0.63	**0.82**	0.53	0.74
	Anxiety	0.53	**0.74**	0.73	0.51	**0.66**	0.65
	Total Score	0.74	0.72	**0.78**	0.69	0.65	**0.77**

*Note: τ*‐correlations of corresponding scale in bold. *100% agreement resulted from the MDD scales of RCADS‐47 and RCADS‐25 being identical. MDD = Major depressive disorder; RCADS = Revised Children's Anxiety and Depression Scale.

Normal and clinically relevant values based on T‐scores for MDD, Anxiety, and Total Scores of RCADS‐47, RCADS‐25, and RCADS‐11 were strongly related, with substantial to perfect observed agreement (0.63 ≤ Cohen's *κ* ≤ 1.0). For depressive symptoms, T‐scores demonstrated between 94.4% and 100.0% consistency between normal and clinically relevant values across all RCADS versions. Anxiety T‐scores were consistent in 90.6%–94.3% of cases, while Total Score T‐scores were consistent in 93.2%–95.2% of cases. Contingency tables presented in Tables 10 and 11 show the agreement within each scale for the different RCADS versions.

**TABLE 10:** Contingency table of normative data comparing the RCADS‐47 with the RCADS‐25 and RCADS‐11 scales.

			RCADS‐25								RCADS‐11							
			**MDD**								**MDD**							
			Normal		Clinical						Normal		Clinical				
			*n*	**%**	*n*	**%**	χ^2^ (1)		*p*	κ	*n*	**%**	*n*	**%**	χ2 (1)	*p*	κ
RCADS‐47	MDD	Normal	1331	85.2	0	0	1562.00		< 0.001	1.0	1279	81.9	52	3.3	958.37	< 0.001	0.78
		Clinical	0	0	231	14.8					36	2.3	195	12.5			

			**Anxiety**								**Anxiety**							
			Normal		Clinical						Normal		Clinical				
			*n*	**%**	*n*	**%**	χ^2^ (1)		*p*	κ	*n*	**%**	*n*	**%**	χ2 (1)	*p*	κ
RCADS‐47	Anxiety	Normal	1268	81.2	36	2.3	944.99		< 0.001	0.78	1275	81.6	29	1.9	969.49	< 0.001	0.79
		Clinical	57	3.6	201	12.9					59	3.8	199	12.7			

			**Total Score**								**Total Score**							
			Normal		Clinical						Normal		Clinical				
			*n*	**%**	*n*	**%**	χ^2^ (1)		*p*	κ	*n*	**%**	*n*	**%**	χ2 (1)	*p*	κ
RCADS‐47	Total Score	Normal	1277	81.8	37	2.4	1050.19		< 0.001	0.82	1274	81.6	40	2.5	986.39	< 0.001	0.80
		Clinical	38	2.4	210	13.4					45	2.9	203	13.0				

*Note:* RCADS = Revised Children's Anxiety and Depression Scale; *n* = sample size; MDD = Major depressive disorder.

**TABLE 11:** Contingency table of normative data comparing the RCADS‐25 and RCADS‐11 scales.

	RCADS‐11								
			MDD*						
			Normal		Clinacal				
			*n*	**%**	*n*	**%**	χ^2^ (1)	*p*	κ
RCADS‐25	MDD	Normal	1279	81.9	52	3.3	958.37	< 0.001	0.78
		Clinical	36	2.3	195	12.5			

			Anxiety						
			Normal		Clinacal				
			*n*	**%**	*n*	**%**	χ^2^ (1)	*p*	κ
RCADS‐25	Anxiety	Normal	1256	80.4	69	4.4	617.55	< 0.001	0.63
		Clinical	78	5.0	159	10.2			

			Total score						
			Normal		Clinacal				
			*n*	**%**	*n*	**%**	χ^2^ (1)	*p*	κ
RCADS‐25	Total score	Normal	1264	80.9	51	3.3	863.39	< 0.001	0.74
		Clinical	55	3.5	192	12.3			

*Note: n* = sample size; MDD = Major depressive disorder; RCADS = Revised Children's Anxiety and Depression Scale.

## 4 Discussion

This study aimed to assess the reliability and validity of two short forms of the RCADS, comprising 25 and 11 items, in a representative German school sample of children and adolescents aged eight to 17 years, and to provide normative data for these instruments. High internal consistency was observed. CFAs for both versions indicated sufficient to good model fit for the two‐factor and higher‐order model, while MG‐CFAs demonstrated measurement invariance across two groups of children and adolescents differently affected by chronic condition. Construct validity was further supported by significant negative correlations with HRQoL and significant positive correlations with functional impairment. Known‐groups validity was demonstrated by higher scores in girls and, in some cases, increasing scores with age. Age‐ and sex‐specific, as well as gender‐unspecific, normative data are provided for both short versions of the RCADS. In direct comparison, the RCADS‐25 exhibited higher internal consistency, whereas the RCADS‐11 indicated a better fit to the data in the CFA. Both instruments produced nearly identical results for convergent and known‐groups validity. Significant, high positive correlations between all corresponding scales of RCADS‐47, RCADS‐25, and RCADS‐11 confirmed that the different forms capture the same constructs, albeit with varying amounts of reliability. T‐scores for MDD, Anxiety, and Total Scores across all RCADS versions identified similar groups individuals with normal and clinically relevant values.

### 4.1 Item and Scale Properties

Good internal consistency has been demonstrated for the German RCADS‐25 and RCADS‐11, supporting the suitability of using these short versions in a German (school) sample. As expected, reliability was slightly lower than that of the German 47‐item version (Grothus et al. 2023), as larger scales often perform better (Tavakol and Dennick 2011). For the RCADS‐11, data from a British sample demonstrated slightly higher reliability than the present study (Radez et al. 2021). This difference may be because the British participants were between 11 and 18 years old, whereas the present sample included children aged eight to 17 years, with the scales revealing poorer reliability for younger children. In particular, RCADS‐11 internal consistency for MDD was unsatisfactory for 8‐ to 9‐year‐olds. A future study focusing on younger children aged eight to 11 years would help evaluate the suitability of the RCADS‐11 in this age group, as short assessment tools are particularly beneficial for younger children, whose reading skills are not fully developed and whose attention spans are shorter than older children. So far, the MDD scale of the RCADS‐11 should be used with caution for children younger than 10 years. The means of the German RCADS‐25 MDD and Anxiety scales are lower than those reported in the original US study by Ebesutani et al. (2012) and in a Spanish‐speaking sample from El Salvador (Young et al. 2021), whereas a Swedish sample found comparable means (Carlander et al. 2024). This highlights the importance of deriving nation‐specific and context‐specific normative data.

### 4.2 Construct Validity

Sufficient to good model fit confirms the 2‐factor structure of the RCADS‐11 and the higher‐order structure of the RCADS‐25. Likewise, there was a strong association between items and factors. The model fit of the 2‐factor model of the RCADS‐25 is in line with findings from other validation studies (Carlander et al. 2024; Lisøy et al. 2022; Young et al. 2021), whereas no other study has yet tested the factor structure of the RCADS‐11 using CFA.

MG‐CFAs demonstrated measurement invariance between two groups of children and adolescents differently affected by chronic disease. Testing measurement invariance is essential to ensure that a set of items measures the same construct across different populations, groups, or conditions, making it a crucial step in establishing factorial validity. Measurement invariance across sex (Buil et al. 2023; Donnelly et al. 2019; Lisøy et al. 2022; Young et al. 2021), age groups (Mathyssek et al. 2013), and different ethnicities, cultures, and countries (Buil et al. 2023; Cervin et al. 2022; Stevanovic et al. 2017; Trent et al. 2013) has already been examined for the RCADS and its short versions. However, to date, measurement invariance of the RCADS has not been tested in populations differently affected by a chronic disease. Due to their chronic condition, pediatric chronic pain patients generally report more symptoms of anxiety and depression than community samples (Zernikow et al. 2012). It is therefore essential that these differences in symptom level are not due to measurement differences. The MG‐CFA for both the RCADS‐25 and the RCADS‐11 confirmed that factor loadings and intercepts were consistent across the two groups. This indicates that the items assess the same construct, and thus, group differences can be interpreted as true differences in the extent of anxiety and depression symptoms in healthy children versus those with chronic pain. This is especially important given that the validation process for the German RCADS‐47 (Grothus et al. 2023; Stahlschmidt et al. 2019) unexpectedly found that the mean anxiety score was lower in a pediatric chronic pain sample compared with a school sample. It can be concluded that anxiety and depressive symptoms measured with the RCADS‐25 and the RCADS‐11 are understood and interpreted similarly across groups, so findings can be generalized. However, further studies on measurement invariance in different groups, especially for the RCADS‐11, are needed as most studies on measurement invariance focus on the 47‐item full version of the RCADS (Buil et al. 2023; Cervin et al. 2022; Donnelly et al. 2019; Mathyssek et al. 2013; Stevanovic et al. 2017; Trent et al. 2013).

### 4.3 Convergent Validity

In line with the validation study for the RCADS‐47, the results for convergent validity were as expected, with significant negative τ‐correlations between the RCADS‐25 and the RCADS‐11 scales and HRQoL, as well as significant positive τ‐correlations between the RCADS scales and functional impairment.

### 4.4 Known‐Groups Validity

Significant sex differences were observed in the MDD, Anxiety, and Total Scores for both the RCADS‐25 and RCADS‐11, with consistently higher means in girls. These findings align with the results from the original German RCADS‐47 item validation (Grothus et al. 2023) as well as several international validation studies for the RCADS long form (Chorpita et al. 2000; Donnelly et al. 2019; Esbjørn et al. 2012; Fard et al. 2021; Giannopoulou et al. 2022; Kösters et al. 2015; Lu et al. 2021; Muris et al. 2002; Ross et al. 2002). For the short versions, however, sex differences have not yet been examined. In accordance with the RCADS‐47 validation (Grothus et al. 2023), anxiety and depressive symptoms increased slightly, but significantly, with age in girls for both the RCADS‐25 and RCADS‐11. In contrast, this association was weakly negative in boys for the RCADS‐25 only. This diverges from the increase in symptoms of anxiety and depression in both sexes described in previous literature (Bitsko et al. 2022; Ghandour et al. 2019). Further research is needed on this topic.

### 4.5 Normative Data

This is the first study to establish normative data for the RCADS‐11 in any language and for the RCADS‐25 in a German sample. Age groups were used instead of grade levels, consistent with the German normative data for the RCADS‐47, as age groups better reflect developmental status in Germany, whereas grade levels can vary in age by up to four years (see Grothus et al. 2023). The mean values in the present sample, stratified by age and sex, are nearly identical to those in a Swedish sample (Carlander et al. 2024), while a Spanish sample from El Salvador (Young et al. 2021) and a US sample primarily showed higher means (Ebesutani et al. 2012). This difference may be explained by cultural factors, such as varying environmental stress due to socioeconomic factors or differences in reporting mental health issues within (Latin‐)American populations. As for the RCADS‐47 (Grothus et al. 2023), calculating T‐scores stratified by age and sex is recommended for the short forms due to the differences in these factors identified in this study. In response to increasing diversity, non‐specific gender T‐scores were also generated, allowing for a gender‐neutral interpretation for practitioners where appropriate.

### 4.6 Comparison of RCADS‐47, RCADS‐25, and RCADS‐11

To evaluate whether the full version and the two short versions of the RCADS measure the same constructs, τ‐correlations were calculated for the MDD, Anxiety, and Total Scores across the RCADS‐47, RCADS‐25, and RCADS‐11. In general, there was a strong association between the three corresponding scales of each version.

Likewise, comparing T‐scores across the three versions demonstrated strong consistency in identifying the same individuals with normal or clinically relevant values. This consistency ensures a high degree of comparability among the instruments and offers practitioners flexibility in choosing the short form that is most appropriate for specific contexts without compromising the quality of the screening results. However, it is recommended to consistently use one of the two short versions rather than switching between them, as the agreement between the RCADS‐25 and the RCADS‐11 was lower than that between the two short versions and the full version.

## 5 Limitations

Despite promising results, several limitations must be acknowledged. First, data from primary school children were collected during the Covid‐19 pandemic in 2021, so our findings should be further examined with a sample of schoolchildren aged eight to 10. Second, the sample size for the 16 to 17‐year‐olds was relatively small. Third, the reliability and validity of the German RCADS‐25 and RCADS‐11 were examined using the same sample and item pool as the validation study for the German RCADS‐47 (Grothus et al. 2023). The additional items not included in the analyses may have introduced systematic bias in responses to the questions used in the short versions. These results should be confirmed with an independent German school sample that completes only the 11 or 25 items of the relevant short version. Fourth, this study did not collect data on anxiety and depression diagnoses. Therefore, clinically relevant cut‐off points for anxiety and depression based on the two RCADS short versions could not be determined. Finally, further reliability and validity measures, such as test‐retest reliability, divergent validity, and sensitivity to change, are necessary for all German RCADS forms.

## 6 Conclusion

The 25‐ and 11‐item versions of the German RCADS are reliable and valid instruments for assessing anxiety and depressive symptoms in children and adolescents aged eight to 17. These shorter versions are advantageous when general anxiety and depressive symptoms need to be measured without specifying subtypes of anxiety, especially in contexts where survey time is limited, such as epidemiological studies, primary care settings, or clinical long‐term assessments. Using these short versions reduces respondent burden, supports early detection of emotional distress, and facilitates timely help‐seeking. Despite the promising results obtained, it must be considered that using the short versions decreases reliability, and subscales of anxiety cannot be assessed. Further research is needed to confirm the diagnostic utility of the RCADS short forms, particularly in clinical samples, and to define clinically relevant cut‐offs.

## Author Contributions

**Susanne Grothus:** conceptualization, data curation, formal analysis, methodology, project administration, investigation, writing – original draft. **Ariane Sommer:** methodology, data curation, investigation, project administration, formal analysis, writing – review and editing. **Benedikt B. Claus:** data curation, formal analysis, writing – review and editing. **Lorin Stahlschmidt:** supervision, data curation, writing – review and editing. **Lea Höfel:** data curation, writing – review and editing. **Bruce F. Chorpita:** data analysis, writing – review and editing. **Julia Wager:** conceptualization, supervision, investigation, formal analysis, project administration, writing – review and editing.

## Conflicts of Interest

We have no known conflicts of interest to disclose.

## Supporting information

Supporting Information S1

## Data Availability

Data for the validation study were retrieved from a study supported by the Joint Federal Committee Innovation Fund (identification number: 01VSF18020). Dr. Chorpita receives funding for support of the RCADS through a contract with the National Institutes of Health (#75N95022C00018). No additional funding was received. The funding sources had no role in the study design; in the collection, analysis, or interpretation of data; in manuscript preparation; or in the decision to submit the article for publication.
