# Supplementary material for: Short Forms of the German Revised Children's Anxiety and Depression Scale (RCADS)–Validation and Normative Data of the 11‐ and 25‐Item Versions
Source: Int J Methods Psychiatr Res. 2025 Apr 26;34(2):e70022. doi: 10.1002/mpr.70022 (PMC12032886; doi:10.1002/mpr.70022)
Supplement: Supplementary file 1 — Supporting Information S1 [file MPR-34-e70022-s001.pdf]

Appendix

Table S1

Cronbach's α, item mean, mean inter-item correlation, and item properties for the higher-order model with anxiety subscales of RCADS-25 item version

Subscale (Cronbach's α)	Item mean (MIC)	Item # and Abbreviation (Item # RCADS-47)	*M*	*SD*	Skewness	Kurtosis	Item-total-correlations	λ higher order model (*N* = 1562)
MDD (.87)	0.67 (0.41)	1 feels sad or empty (2)	0.77	0.80	0.88	0.29	.65	.78
		4 feels nothing is much fun anymore (6)	0.41	0.70	1.78	2.77	.60	.73
		8 has trouble sleeping (11)	0.82	0.89	0.92	0.08	.55	.65
		10 has problems with appetite (15)	0.57	0.78	1.33	1.24	.48	.59
		13 has no energy (19)	0.62	0.73	1.11	1.07	.65	.74
		15 cannot think clearly (25)	0.70	0.70	0.82	0.67	.60	.72
		16 feels worthless (29)	0.50	0.80	1.57	1.68	.63	.85
		19 feels like doesn't want to move (40)	0.63	0.83	1.22	0.77	.59	.72
		21 feels tired a lot (21)	0.98	0.90	0.66	-0.33	.58	.65
		24 feels restless (47)	0.73	0.81	0.98	0.41	.58	.71

Subscale (Cronbach's α)	Corresponding subscale of RCADS-47	Item mean (MIC)	Item # and Abbreviation (Item # RCADS-47)	*M*	*SD*	Skewness	Kurtosis	Item-total-correlations	ʎ higher order model (*N* = 1562)
Anxiety (α=.87) M_{item} =0.65 (MIC=0.32)	GAD	1.00 (0.44)	5 worries something awful will happen to family (13)	1.37	1.01	0.31	-0.98	.53	.70
			18 thinks about death (37)	0.89	0.90	0.78	-0.23	.45	.70
			25 worries something bad will happen (27)	0.73	0.80	1.00	0.59	.60	.80
	OCD	0.45 (0.41)	12 has to do things over and over again (42)	0.68	0.94	1.22	0.40	.43	.59
			17 has to think special thoughts to stop bad events (31)	0.25	0.60	2.64	7.12	.46	.80
			23 has to do things the right way to stop bad events (44)	0.43	0.71	1.73	2.68	.57	.87
	PD	0.42 (0.45)	11 suddenly becomes dizzy for no reason (36)	0.49	0.75	1.46	1.42	.57	.76
			14 suddenly starts shaking for no reason (26)	0.48	0.77	1.57	1.73	.53	.70
			20 worries of suddenly getting scared for no reason (41)	0.30	0.62	2.35	5.76	.50	.85
	SAD	0.33 (0.26)	3 feels afraid of being alone at home (5)	0.39	0.73	2.02	3.62	.39	.60
			6 afraid of being in crowded places (33)	0.30	0.63	2.37	5.69	.21	.81
			9 feels scared to sleep alone (17)	0.30	0.62	2.42	6.34	.41	.60
	SP	1.05 (0.51)	2 worries when done poorly at something (4)	1.36	0.91	0.30	-0.70	.51	.72
			7 worries what others think of them (32)	0.90	0.92	0.80	-0.23	.64	.81
			22 afraid of looking foolish (43)	0.90	0.88	0.79	-0.09	.61	.79

Note. *M* = mean; *SD* = standard deviation; MIC = Mean inter-item correlation; MDD = Major depressive disorder; GAD = Generalized anxiety disorder; OCD = Obsessive-compulsive disorder; PD = Panic disorder; SAD = Separation anxiety disorder; SP = Social phobia.

Table S2

Cronbach's α, item mean, mean inter-item correlation, and item properties for the 2-factor model of RCADS-11 item version

Subscale (Cronbachs α)	Item mean (MIC)	Item # and Abbreviation (Item # RCADS-47)	*M*	*SD*	Skewness	Kurtosis	Item-total-correlations	λ 2-Factor model
MDD (.81)	0.66 (0.47)	2 has no energy (19)	0.62	0.73	1.11	1.07	.63	.76
		5 feels nothing is much fun anymore (6)	0.41	0.70	1.78	2.77	.59	.74
		7 feels worthless (29)	0.50	0.80	1.57	1.68	.61	.86
		8 feels sad or empty (2)	0.77	0.80	0.88	0.29	.65	.82
		10 feels tired a lot (21)	0.98	0.90	0.66	-0.33	.52	.65
Anxiety (.79)	0.56 (0.40)	1 trouble going to school (18)	0.29	0.60	2.30	5.60	0.51	.70
		3 worries to go to bed at night (45)	0.41	0.71	1.81	2.86	0.55	.72
		4 worries about what is going to happen (35)	0.71	0.79	1.02	0.67	0.61	.76
		6 suddenly feels scared for no reason (34)	0.30	0.62	2.22	4.86	0.56	.76
		9 when has a problem, heart beats fast (24)	0.87	0.90	0.84	-0.09	0.53	.65
		11 worries might look foolish (20)	0.78	0.85	0.91	0.15	0.54	.71

Note. *M* = mean; *SD* = standard deviation; MIC = Mean inter-item correlation; MDD = Major depressive disorder.

Table S3

Ranges, means, and standard deviations for the RCADS-25 scales stratified by age and sex

Sex	Age	Scale	*N*	Minimum	Maximum	*M*	*SD*
**Girls**	8-9 years	MDD	111	0	22	7.37	4.60
		Anxiety	111	0	30	10.41	6.33
		Total Score	111	0	50	17.86	10.34
	10-11 years	MDD	185	0	27	6.32	5.85
		Anxiety	185	0	38	10.30	7.65
		Total Score	185	0	65	16.63	12.85
	12-13 years	MDD	227	0	30	7.80	6.12
		Anxiety	227	1	40	10.63	7.45
		Total Score	227	1	70	18.42	12.84
	14-15 years	MDD	225	0	29	9.40	5.97
		Anxiety	225	1	34	11.37	6.64
		Total Score	225	1	63	20.77	11.66
	16-17 years	MDD	70	1	29	10.06	6.34
		Anxiety	70	0	29	11.49	7.11
		Total Score	70	3	55	21.54	12.26
**Boys**	8-9 years	MDD	103	0	16	7.29	3.83
		Anxiety	103	0	24	8.61	5.61
		Total Score	103	0	40	15.90	8.58
	10-11 years	MDD	235	0	23	5.06	4.32
		Anxiety	235	0	28	7.77	5.58
		Total Score	235	0	48	12.83	9.18

**Sex**	**Age**	**Scale**	***N***	**Minimum**	**Maximum**	***M***	***SD***
**Boys**	12-13 years	MDD	177	0	19	4.47	3.94
		Anxiety	177	0	20	6.32	4.68
		Total Score	177	0	38	10.80	7.86
	14-15 years	MDD	167	0	20	5.10	4.19
		Anxiety	167	0	38	6.16	5.15
		Total Score	167	0	58	11.26	8.44
	16-17 years	MDD	62	0	21	5.40	5.03
		Anxiety	62	0	21	6.50	4.72
		Total Score	62	0	36	11.90	9.03

Note. RCADS = Revised Children's Anxiety and Depression Scale; *N* = sample size; *M* = mean; *SD* = standard deviation; MDD = Major depressive disorder.

Table S4

Ranges, means, and standard deviations for the RCADS-11 scales stratified by age and sex

**Sex**	**Age**	**Scale**	***N***	**Minimum**	**Maximum**	***M***	***SD***
**Girls**	8-9 years	MDD	111	0	8	3.30	2.09
		Anxiety	111	0	14	3.90	3.31
		Total Score	111	0	22	7.20	4.93
	10-11 years	MDD	185	0	14	2.91	3.08
		Anxiety	185	0	16	3.66	3.47
		Total Score	185	0	29	6.57	6.14
	12-13 years	MDD	227	0	15	3.87	3.36
		Anxiety	227	0	18	4.19	3.65
		Total Score	227	0	33	8.05	6.56
	14-15 years	MDD	225	0	15	4.98	3.43
		Anxiety	225	0	17	4.35	3.33
		Total Score	225	0	32	9.33	6.08
	16-17 years	MDD	70	1	15	5.49	3.51
		Anxiety	70	0	16	4.79	3.76
		Total Score	70	1	31	10.27	6.69
**Boys**	8-9 years	MDD	103	0	9	3.29	1.94
		Anxiety	103	0	10	2.72	2.34
		Total Score	103	0	16	6.01	3.75
	10-11 years	MDD	235	0	13	2.26	2.27
		Anxiety	235	0	14	2.88	2.77
		Total Score	235	0	25	5.13	4.56
	12-13 years	MDD	177	0	10	2.02	2.07
		Anxiety	177	0	10	2.20	2.24
		Total Score	177	0	19	4.23	3.81

Sex	Age	Scale	*N*	Minimum	Maximum	*M*	*SD*
**Boys**	14-15 years	MDD	167	0	10	2.56	2.40
		Anxiety	167	0	14	2.25	2.30
		Total Score	167	0	22	4.80	4.16
	16-17 years	MDD	62	0	12	2.79	2.78
		Anxiety	62	0	10	2.68	2.62
		Total Score	62	0	20	5.47	4.93

Note. RCADS = Revised Children's Anxiety and Depression Scale; *N* = sample size; *M* = mean; *SD* = standard deviation; MDD = Major depressive disorder.

Table S5

Ranges, means, and standard deviations for the RCADS-25 scales stratified by age

**Gender**	**Age**	**Scale**	***N***	**Minimum**	**Maximum**	***M***	***SD***
**non-specific**	8-9 years	MDD	214	0	22	7.37	4.26
		Anxiety	214	0	30	9.55	6.05
		Total Score	214	0	50	16.92	9.56
	10-11 years	MDD	420	0	27	5.62	5.08
		Anxiety	420	0	38	8.89	6.68
		Total Score	420	0	65	14.50	11.10
	12-13 years	MDD	404	0	30	6.34	5.52
		Anxiety	404	0	40	8.74	6.72
		Total Score	404	0	70	15.08	11.57
	14-15 years	MDD	392	0	29	7.57	5.69
		Anxiety	392	0	38	9.15	6.57
		Total Score	392	0	63	16.72	11.42
	16-17 years	MDD	132	0	29	7.87	6.20
		Anxiety	132	0	29	9.14	6.57
		Total Score	132	0	55	17.02	11.85

Note. RCADS = Revised Children's Anxiety and Depression Scale; *M* = mean; *N* = sample size; *SD* = standard deviation; MDD = Major depressive disorder.

Table S6

Ranges, means, and standard deviations for the RCADS-11 scales for non-specific gender

Gender	Age	Scale	*N*	Minimum	Maximum	*M*	*SD*
**non-specific**	8-9 years	MDD	214	0	9	3.29	2.01
		Anxiety	214	0	14	3.33	2.93
		Total Score	214	0	22	6.63	4.43
	10-11 years	MDD	420	0	14	2.55	2.67
		Anxiety	420	0	16	3.22	3.12
		Total Score	420	0	29	5.77	5.36
	12-13 years	MDD	404	0	15	3.06	3.01
		Anxiety	404	0	18	3.32	3.26
		Total Score	404	0	33	6.38	5.84
	14-15 years	MDD	392	0	15	3.95	3.26
		Anxiety	392	0	17	3.45	3.11
		Total Score	392	0	32	7.40	5.80
	16-17 years	MDD	132	0	15	4.22	3.45
		Anxiety	132	0	16	3.80	3.43
		Total Score	132	0	31	8.02	6.38

Note. RCADS = Revised Children's Anxiety and Depression Scale; *M* = mean; *N* = sample size; *SD* = standard deviation; MDD = Major depressive disorder.

Table S7

Percentile scores for RCADS-25 Depression scale stratified by age and gender

Percentile	Raw Score RCADS-25 MDD				
	boys 8-9	boys 10-11	boys 12-13	boys 14-15	boys 16-17
**10**	2	0	0	1	0
**20**	3	1	1	2	1
**30**	5	2	2	2	2
**40**	6	3	3	3	2
**50**	8	4	4	4	4
**60**	9	5	5	5	6
**70**	9	7	5	7	7
**80**	11	8	7	9	10
**85**	11	9	9	10	11
**90**	12	10	10	11	14
**95**	14	14	12	13	16
**97.5**	15	15	16	17	19
	**girls 8-9**	**girls 10-11**	**girls 12-13**	**girls 14-15**	**girls 16-17**
**10**	1	1	1	2	4
**20**	3	2	3	4	5
**30**	5	2	4	6	5
**40**	6	4	5	7	7
**50**	6	5	6	9	9
**60**	8	6	8	10	10
**70**	10	8	10	12	12
**80**	12	10	12	14	17
**85**	12	11	13	15	19
**90**	14	15	16	18	20
**95**	15	20	22	22	22
**97.5**	18	24	24	24	24

Note. RCADS = Revised Children's Anxiety and Depression Scale

Table S8

Percentile scores for RCADS-25 Anxiety scale stratified by age and gender

Percentile	Raw Score RCADS-25 Anxiety				
	**boys 8-9**	**boys 10-11**	**boys 12-13**	**boys 14-15**	**boys 16-17**
**10**	2	1	1	1	1
**20**	3	3	2	2	2
**30**	5	4	3	3	3
**40**	7	5	4	4	5
**50**	8	7	5	5	5
**60**	9	8	6	6	7
**70**	11	10	8	7	8
**80**	13	12	10	10	11
**85**	15	13	12	10	13
**90**	17	15	13	13	14
**95**	20	18	16	16	16
**97.5**	22	22	19	18	19
	**girls 8-9**	**girls 10-11**	**girls 12-13**	**girls 14-15**	**girls 16-17**
**10**	3	2	3	3	4
**20**	5	4	4	6	5
**30**	7	5	6	7	6
**40**	8	7	8	9	8
**50**	9	9	9	10	10
**60**	11	10	11	12	14
**70**	14	13	13	14	15
**80**	16	16	16	17	17
**84**	17	18	17	18	20
**90**	19	20	21	20	23
**95**	24	26	24	25	25
**97.5**	26	29	30	29	27

Note. RCADS = Revised Children's Anxiety and Depression Scale

Table S9

Percentile scores for RCADS-11 Depression scale stratified by age and gender

Percentile	Raw Score RCADS-11 MDD				
	boys 8-9	boys 10-11	boys 12-13	boys 14-15	boys 16-17
**10**	1	0	0	0	0
**20**	1	0	0	0	0
**30**	2	1	1	1	1
**40**	3	1	1	1	1
**50**	3	2	1	2	2
**60**	4	2	2	3	3
**70**	4	3	3	4	4
**80**	5	4	3	5	5
**85**	5	5	4	5	6
**90**	6	5	5	6	8
**95**	7	6	6	8	8
**97.5**	8	8	8	8	10
	girls 8-9	girls 10-11	girls 12-13	girls 14-15	girls 16-17
**10**	1	0	0	1	2
**20**	1	1	1	2	3
**30**	2	1	2	3	3
**40**	3	1	2	4	4
**50**	3	2	3	4	4
**60**	4	3	4	5	5
**70**	4	3	5	7	7
**80**	5	5	6	8	9
**85**	6	6	7	9	9
**90**	6	7	9	10	11
**95**	7	10	11	11	13
**97.5**	8	13	13	13	14

Note. RCADS = Revised Children's Anxiety and Depression Scale

Table S10

Percentile scores for RCADS-11 Anxiety scale stratified by age and gender

Percentile	Raw Score RCADS-11 Anxiety				
	boys 8-9	boys 10-11	boys 12-13	boys 14-15	boys 16-17
**10**	0	0	0	0	0
**20**	1	1	0	0	1
**30**	1	1	0	1	1
**40**	1	2	1	1	1
**50**	2	2	2	2	2
**60**	3	3	2	2	3
**70**	4	4	3	3	3
**80**	5	5	4	4	4
**85**	5	6	4	5	5
**90**	6	7	5	5	8
**95**	7	8	7	7	9
**97.5**	8	9	9	8	9
	**girls 8-9**	**girls 10-11**	**girls 12-13**	**girls 14-15**	**girls 16-17**
**10**	1	0	1	1	1
**20**	1	1	1	2	1
**30**	2	1	2	2	2
**40**	2	2	3	3	3
**50**	3	3	3	4	4
**60**	4	4	4	4	6
**70**	5	5	5	5	6
**80**	6	6	6	7	8
**85**	7	8	7	8	9
**90**	9	9	10	9	10
**95**	10	10	12	12	12
**97.5**	13	13	14	13	14

Note. RCADS = Revised Children's Anxiety and Depression Scale
